# Optimization and characterization of polyhydroxybutyrate produced by *Vreelandella piezotolerans* using orange peel waste

**DOI:** 10.1038/s41598-025-10899-x

**Published:** 2025-07-16

**Authors:** Mahmoud H. Hendy, Amr M. Shehabeldine, Amr H. Hashem, Ahmed F. El-Sayed, Hussein H El-Sheikh

**Affiliations:** 1 Botany and Microbiology Department, Faculty of Science, Al-Azhar University, Cairo, 11884 Egypt; 2 Microbial Genetics Department, Biotechnology Research Institute, National Research Centre, Giza, Egypt; 3 Egypt Center for Research and Regenerative Medicine (ECRRM), Cairo, Egypt

**Keywords:** Polyhydroxybutyrate, Response surface optimization, *Vreelandella piezotolerans*, Halophilic bacteria., Biotechnology, Microbiology

## Abstract

Microorganisms are promising, cost-effective, and sustainable producers of bioproducts, including polyhydroxybutyrate (PHB), a biodegradable polymer that offers an eco-friendly alternative to synthetic plastics. This study investigates PHB production using the newly isolated *Vreelandella piezotolerans* with orange peel waste as the substrate. Notably, this is the first research to optimize PHA production with *V. piezotolerans*, utilizing Design Expert 7.0 software. Various bacterial isolates were screened, and the most efficient strain, MH46, was identified as *V. piezotolerans* through 16 S rRNA sequencing and registered in GenBank (accession number PP826285). Single-factor optimization was conducted to determine optimal fermentation conditions. The results of the single-factor optimization were used to conduct Plackett-Burman design experiments. The response surface optimization was then completed. Results revealed that temperature, agitation rate, and inoculum size significantly influence PHA production. The extracted PHB was characterized using GC-MS, NMR, FTIR, XRD, and thermal analysis. This study is the first to report PHB accumulation by *V. piezotolerans* using orange peel waste as the sole carbon source.

## Introduction

Plastics made from fossil fuels are essential to the lifestyle that we live today^1^. Artificial plastics have a major detrimental effect on ecosystems, ecology, biota, economics, and health of human^2^. As a result, there is an increasing demand for bioplastics that provide alternative life cycles and sources of raw materials, such as polyhydroxyalkanoates (PHA), polylactic acid, biopolypropylene, and biopolyamide^3^. In industrial applications and waste management, Polyhydroxybutyrate represents a promising and environmentally friendly alternative^4^. PHAs were accumulated by microorganisms as granules that serve as an alternative source of energy as well as carbon storage. In culture media, PHA accumulation usually occurs when there are abundant carbon sources but limited nitrogen sources^5,6^. PHAs are a better alternative to bioplastic than petroleum-based polymers. The biocompatibility and biodegradability of PHAs bioplastics enable a range of uses in both agriculture and medicine^7^. Lemoigne initially discovered polyhydroxybutyrate (PHB), a form of PHA that is currently the subject of extensive research, in 1925^8^. A vast range of resources are available in marine habitats for the production of macromolecules with industrial and biological significance, including hydrolytic enzymes, biofuels, and pharmaceuticals^9^. Due to environmental stresses like the marine environment’s low temperature and elevated salt concentration, marine microorganisms have adapted to extreme conditions. This has allowed strong survivors with competitive properties like cryophilicity or salt tolerance to be selected for the production of macromolecules^10,11^. Consequently, they may be utilized to develop an economical bioprocess that does not require some necessary manufacturing steps, such as water treatment, sterilization or sterilization. Numerous investigations have looked into the possibility of genetically modifying halotolerant bacterial strains to increase their capacity to produce PHA^12^, and using them in industrial biotechnology as such^13^. This results in the cost-effectiveness of the substrates employed as the carbon source in PHB production. Thus, the most crucial strategy for reducing production costs is to use cheap raw materials^14^. For this reason, to make biopolymer-producing microorganisms financially viable, it is preferred to culture them using inexpensive raw resources, such as agricultural and agro-industrial residues^15^. Orange peel waste represents a valuable nutrient source for bacteria that produce polyhydroxybutyrate^16^, due to its composition includes soluble and insoluble carbohydrates and relatively low protein content^17^. This results in a favorable carbon-to-nitrogen ratio. Additionally, with an estimated global production of 10 million tons annually, orange peel waste constitutes the primary byproduct of the citrus processing industry^18,19^. Traditional optimization methods often fail to consider molecular interactions between variables, which can lead to inaccurate identification of optimal production conditions. These methods also tend to be time-consuming and labor-intensive^20^. In contrast, Response Surface Methodology (RSM) is an effective alternative that is now widely applied to optimize the production of target metabolites^21^. It allows for the simultaneous analysis of multiple factors and their relative contributions. Ignoring interactions among variables can result in misjudging the true impact of individual factors, either by overstating or understating their effects^22^. Furthermore, PHB production can be improved by optimizing procedure factors utilizing a variety of tools for optimization^23^. Previous research has used Response Surface Methodology (RSM) to enhance the production rate of PHAs by various bacteria, including *Rhodobacter sphaeroides*, *Bacillus coagulans*, and *Ralstonia eutropha*. Utilization is readily available, and low-cost substrates have promoted the current increase in Gram-positive bacteria stains. in optimizing bacterial requirements for optimal PHA production^24^. By screening and examining the interactions between the parameters, a factorial design employing RSM is utilized to show the cumulative effect of the factors. Regarding low-cost carbon sources, statistical optimization approaches remain a crucial tactic for achieving an ideal PHA concentration immediately before large-scale manufacturing^25^.

In this study, PHB was produced by cultivating *V. piezotolerans* in artificial media containing orange peel. The PHB derived from the media was thoroughly characterized utilizing advanced analytical techniques including^1^H and ^13^C nuclear magnetic resonance (NMR) spectroscopy, Fourier transform infrared (FTIR) spectroscopy, gas chromatography-mass spectrometry (GC-MS), X-ray diffraction (XRD), and thermogravimetric analysis (TGA). The ultimate goal is to reduce the production costs associated with PHB and overcome the barriers to its industrialization, thereby contributing to sustainable ecological management through biotechnological applications. The ultimate objective is to lower PHB production costs and remove barriers to its industrialization, which will enable biotechnological applications to support sustainable ecological management.

## Materials and methods

### Isolation sources and medium

Different samples were collected from different sites in Egypt as follows: solid samples included soil from near hot springs, salt water, and compost. Liquid samples include saltwater, seawater, and hot wells. To screen marine bacteria, a modified Tryptone Yeast Extract Saline (TYS) Medium was used, and it contained (g/L)^26^: NaCl 75; KCl 0.7; CaCl_2_⋅2H_2_O 1.4; MgSO_4_⋅7H_2_O 6.8, MgCl_2_⋅6H_2_O 5.4; NaHCO_3_ 0.2; yeast extract 0.5, and peptone 1. TYS medium enriched with 10% NaCl, 20 g/L glucose and sucrose, and 500 g/L orange peel extract was used as the carbon source. 1 N HCl and 1 N NaOH were utilized to adjust the medium’s pH to 7.0.

### Screening tests for PHB-producing isolates

For the initial screening of bacteria that produce PHB, the following procedures were conducted: 0.5 µg of Nile Red (Sigma-Aldrich, Germany) per mL of solid medium was employed. The viable colony staining technique was prepared and sterilized before being placed into Petri dishes. After three days of culture at 37 °C and subsequent streaking on agar plates, the isolates obtained were examined under a UV lamp. Positive results were reported for the illuminated colonies^27^. A final pH of 7.0 was maintained for one day on a nutrient agar plate at 37 °C (Himedia Laboratories, India) to preserve the pure cultures of bacteria that produce PHA for future use. A confirmation screening test for the isolates was performed utilizing Sudan Black-B (SBB) stain (Sigma-Aldrich, Germany) to assess PHA production^28^. A 2% sucrose supplement was added to the TYS medium, which was employed to cultivate a subset of bacterial isolates for secondary screening. The medium was incubated at 37 °C and 150 rpm for 72 h. Measurements of the polymers were conducted after extraction.

### Preparation of orange peel waste (OPW) extract

OPW was gathered from Giza, Egypt’s local marketplaces. After being collected, the samples were taken to the lab and processed right away. After choosing OPW, who showed no signs of illness, they were given two rounds of distilled water (DW) washings. After cutting the peel into small pieces (about 1 cm), 500 g was put in a 1 L conical flask with 1000 mL of deionized water. The mixture was then blended at 1000 rpm for three minutes using a blender (mixer). The mixture was then filtered through Whatman no. 1 filter paper, collected in a sterilized bottle, and stored at 4 °C until it was needed^29,30^. The main ingredients of OPW are lignin, cellulose, hemicellulose, pectin, and total sugars^31^. The fiber content (cellulose, hemicellulose, and lignin) reported by Marín et al.,^32^. Orange peel waste (OPW) is a lignocellulosic material rich in pectin (up to 42.7%)^33^.

### Identification of the most potent bacterial isolation

#### Biochemical and morphological characteristics

To identify the most potent isolation MH 46, the morphological (shape, Gram’s reaction) and some biochemical characteristics were examined.

Genomic DNA was isolated from the overnight culture of chosen isolates using a Qiagen DNA purification kit, according to the manufacturer’s guidelines (Qiagen, Hilden, Germany). PCR amplification of 16 S rRNA was conducted using genomic DNA from the isolates as a template. Two primers were utilized: 8 F (5’-AGAGTTTGATCCTGGCTCAG-3’) and 1495 R (5’-CTACGGCTACCTTGTTACGA-3’), along with GoTaq Flexi DNA Polymerase (Promega, WI, USA), following the protocol established by^34^. A total reaction volume of 50 µL was achieved by combining 10 µL of 5× Go Taq Flexi buffer, 0.5 µL of Go Taq Flexi DNA polymerase, 2 µL of 25 mM MgCl₂, 1 µL of 10 mM PCR nucleotide mix, 1.5 µL of double-distilled water, and 1 µL of DNA. The PCR cycling conditions included an initial denaturation step at 95 °C for 5 min, followed by 35 cycles of denaturation at 95 °C for 1 min, annealing at 50 °C for 1 min, and extension at 72 °C for 2 min. The reaction concluded with a final extension at 72 °C for 10 min, followed by storage at + 4 °C. The amplified 16 S rRNA fragments were sequenced at the HVD sequencing service in Germany. The resulting sequences were edited and shortened using BioEdit version 7.2.5. Subsequently, these modified sequences were aligned with those in the GenBank database through BLAST analysis. A phylogenetic tree was constructed based on the 16 S rRNA sequences using the neighbor-joining method, facilitating the identification of closely related bacterial species by employing molecular evolutionary genetics approaches^35,36^.

### Factors affecting PHB production (single factor optimization)

This experiment investigated the impact of salinity, concentration of the nitrogen supply, concentration of orange peel, and concentration of peptone and yeast extract to optimize the culture medium. Table 1 lists the specifics of the various settings. Following fermentation, the bacteria were analyzed to calculate the PHB, and the DCW and PHB yields were used to identify the ideal single-factor conditions. The PHA recovery yield is calculated using the formula :

1 $${\text{PHA}}~{\text{Recovery}}~{\text{Yield}}~\left( \% \right) = \left( {\frac{{Weight~of~PHA~obtained~\left( {g/L} \right)}}{{Intial~dry~Weight~of~biomass~\left( {g/L} \right)}}} \right)~ \times {\text{1}}00$$

To maximize fermentation conditions, the effects of temperature, initial pH, agitation speed, incubation time, and inoculation size were also investigated.

**Table 1:** Gradient setting of each factor in the single-factor optimization experiment.

Concentration gradient setting												
	Orange peel (g/L)		100	200	300	400	500	600	700	800	900	1000
Single-factor optimization for media	Type of nitrogen source (g/L)	NH_4_Cl	1.5	–	–	–	–	–	–	–	–	–
		(NH_4_)_2_SO_4_	1.5	–	–	–	–	–	–	–	–	–
		(NH_4_)_2_HPO_4_	1.5	–	–	–	–	–	–	–	–	–
		NaNO_3_	1.5	–	–	–	–	–	–	–	–	–
		Casein	1.5	–	–	–	–	–	–	–	–	–
		Peptone	1.5	–	–	–	–	–	–	–	–	–
		Yeast extract	1.5	–	–	–	–	–	–	–	–	–
		Peptone & yeastextract (2:1 ratio respectively (	1.5	–	–	–	–	–	–	–	–	–
Fermentation condition single-factor optimization	Peptone &yeast extract (g/L) (2:1 ratio respectively (		0.5	1	1.5	2	2.5	3	4	–	–	–
	Incubation time (h)		12	24	36	48	60	72	84	96	–	–
	Inoculation size (%) (v/v)		1	2	4	6	8	10	12	-	–	–
	Initial pH		5	6	6.5	7	7.5	8	9	10	–	–
	Salinity (%)		0	2	5	7.5	12.5	15	17.5	20	–	–
	Temperature (^o^C)		25	30	35	37	40	45	50	–	–	–
	Agitation rate (rpm)		0	50	150	200	250	–	–	–	–	–

#### Optimization of PHB using Plakett–Burman (PB) design

Screening was done for the significant factors affecting PHB production using the PB experimental design^37^. As illustrated in Table 2, eleven variables related to medium composition and culture conditions were analyzed at low (− 1) and high (+ 1) levels. 12 variables (*n* + 1) can be investigated due to the two-level factorial design of the PB matrix design^38^. To avoid errors, this design used three replicated center points.

2 $$\:Y=\beta\:0+\sum\:\beta\:ixi$$

Y is the anticipated response, β_0_ is the model intercept, β_i_ denotes the linear coefficient, and X_i_ indicates the levels of the independent variables. PHB production was measured in triplicate, and the response was calculated as the average of these values. Variables exhibiting significant effects at the 95% confidence level (*p* < 0.05) were identified as substantially influencing PHB yield and were subsequently utilized for further optimization.

#### Response surface methodology

To determine process stability and inherent variability, a second-order response surface was developed using a Box-Behnken design (BBD), incorporating three factors, three levels, and three replicates at the center point^39^. The Plackett-Burman design was followed in the selection of the center points and specifications. The selection of center points and specifications adhered to the Placket-Burman design framework. Table 3 presents the three levels: “high (+ 1),” “middle (0),” and “low (− 1).” The resulting data underwent regression analysis via the “Design Expert” software package (Version 7.0). The precision of the polynomial model was evaluated using the coefficient of determination (R²^2^. Each experimental trial was conducted in triplicate. The second-order polynomial regression model can be expressed as follows:

3 $$Y={\beta _0}+\sum {{\beta _{ixi}}} +\sum {\beta _{{iixi}}^{2}} +\sum {{\beta _{ijxixj}}} +\Sigma$$

In this equation x_i_ and x_j_ are the coded independent variables, Y is the expected response, β_0_ is the intercept, β_i_ is the linear coefficient, β_ij_ represents the interactive coefficients, β_ii_ denotes the quadratic coefficients, and ∑ accounts for the error term.

**Table 2:** Levels of each factor in the Plackett-Burman experimental Design.

Factor code	Factor	Level value	
		Low (− 1)	High (+ 1)
A	Orange peel concentration (g/L)	600	1000
B	Temperature (^o^C)	30	45
C	Sodium chloride (%)	10	15
D	Incubation time (hr.)	48	72
E	Inoculum size (%) (v/v)	2	12
F	Agitation rate (rpm)	100	200
G	Yeast extract (g/L)	0.25	0.75
H	MgSO_4_.7H_2_O (g/L)	1.7	5.1
J	MgCl_2_.6H_2_O (g/L)	1.35	4.05
K	Peptone (g/L)	0.5	1.5
L	Initial pH	6	8

**Table 3:** Levels of each factor in the Box-Behnken experimental design.

Factor	Level value		
	− 1	0	+ 1
Inoculum size (%) (v/v)	6	8	10
Agitation rate (rpm)	120	150	180
Temperature (^o^C)	35	37	40

### Characterization of PHA synthesis by *V. piezotolerans*

#### Gas chromatography-mass spectrometry (GC–MS) detection

Research conducted at the National Research Center in Dokki, Egypt, utilized a direct capillary column (30 m in length, 0.25 μm in thickness, and 25 mm in internal diameter) along with a Trace GC1310-ISQ mass spectrometer (Thermo Scientific, Austin, Texas, USA)^40^. A specified amount of air-dried biomass or pure PHA was placed in a clean glass tube, to which 1 ml of chloroform, 850 µl of methanol, and 150 µl of H2SO4 were added. The tube was sealed and subjected to hydrolysis at 100 °C for 160 min. After hydrolysis, an equal volume of water was incorporated, and the mixture was stirred thoroughly. A 2 µl sample was then extracted from the bottom layer for injection. Benzoic acid was employed as the internal standard.

#### Fourier transform infrared chromatography analysis (FTIR)

The functional groups present in the isolated PHA were analyzed using Fourier Transform Infrared (FTIR) spectroscopy at the National Research Center in Dokki, Egypt. The biopolymer was dissolved in chloroform and mixed with potassium bromide (KBr) pellets, after which the solvent was removed. Infrared spectra for the samples were recorded using a Japanese-made FTIR/4 Jascoo, spanning the wave number range of 400 to 4000 cm^− 1^^41^.

#### Nuclear magnetic resonance analysis (NMR)

Dried samples of PHA (25 mg) were treated with 100 µl of deuterochloroform (CDCl_3_) for NMR analysis. The samples’ chemical structure was examined using a 500 MHz spectrometer ^1^H and ^13^C NMR (300 MHz Varian Mercury Plus NMR Spectrometer)^41^.

#### X-ray diffraction analysis (XRD)

Properly purified PHA samples were placed on a glass slide and compressed into pieces for analysis. XRD data were measured by the modern diffractometer Bruker d8 advance, Germany, using copper source at 40 mA, 40 kV, in the 2θ range 5º-80º and step 0.05º. The intensities were recorded accordingly^42^.

#### Thermogravimetry analysis (TGA)

A sample weighing 5 mg was analyzed using the Themsys One Plus SETARAM (France) under a nitrogen atmosphere, with a nitrogen flow rate of 20 mL/min and a heating rate of 10 °C/min. Differential thermogravimetry (DTG) and TGA were used to investigate the polymer’s breakdown temperature and its thermal stability^43^.

#### Differential scanning calorimetry) DSC)

Glass transition (T_g_) and melting temperature (T_m_) analyses were performed on all polymer samples under N2 using DSC Themsys One Plus SETARAM France. The analysis comprised three temperature cycles: heating from room temperature to 250 °C, cooling from 250 °C to -50 °C, and reheating from − 50 °C to 250 °C. The heating and cooling rates were set at 10 °C/min and 5.0 °C/min, respectively^44^.

### Analytical methods

#### Determination of cell dry weight

The optical density at 600 nm was measured using a spectrophotometer (M-ETKAL-721) to evaluate cell growth. Following a five-minute centrifugation at 10,000 rpm and 4 °C, the cell pellet was washed with distilled water. To ensure constant weight, the pellet was dried overnight at 105 °C. A standard calibration curve correlating OD_600_ with cell dry weight was used to ascertain cell mass concentration^27^.

#### Determination amount of PHA

The extraction of PHA from bacteria was carried out at 10,000 rpm for 10 min and was used for collecting the bacterial cells following the incubation period. The resultant pellet was treated with a 4% w/v sodium hypochlorite solution and incubated for one hour at 37 °C, followed by a 15-minute centrifugation at 5000 rpm. The pellet was then washed with distilled water and cleaned with acetone. A boiling chloroform solution (5 ml) was employed to dissolve the pellet, which was subsequently allowed to evaporate. The extracted PHB was analyzed spectrophotometrically following the method outlined in^45^. The method was performed on the extracted PHB. Ten milliliters of concentrated H_2_SO_4_ were added to the sample containing PHB in chloroform, which was then sealed with a heated glass marble for 10 min in a water bath. After cooling, the solution was transferred to a silica cuvette for analysis using a UV spectrophotometer (JENWAY 6305), measuring absorbance at 235 nm against a sulfuric acid blank. A standard curve was established using PHB concentrations ranging from 0.5 to 3.5 mg/ml.

### Statistical analysis

All experiments were conducted in triplicate, with means and standard deviations reported for each set of replicates. A stepwise regression approach was employed to formulate an optimal model, resulting in an ideal regression equation. The main effects of the fitting model were analyzed using ANOVA, with significance defined as *P* < 0.05 to mitigate type I error rates. Data analysis was performed using Design Expert 7.0 and Excel 365.

## Results and discussion

### Isolation and screening of PHB-producers from different sources

**Fig. 1:**
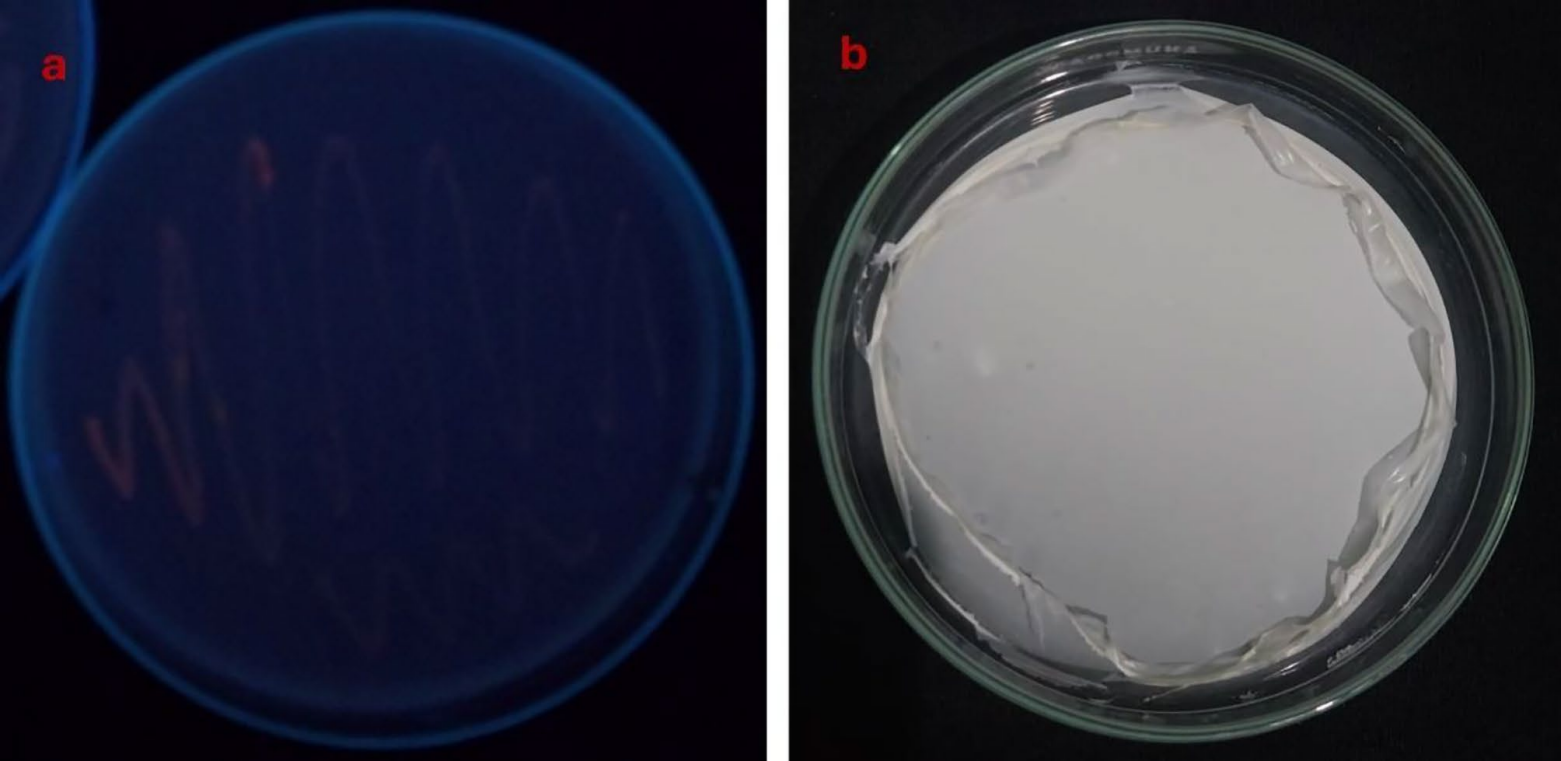
(**a**) shows Nile red staining of bacterial isolate MH 46 under UV light, and (**b**) shows PHA after extraction from strain MH 46.

As mentioned in the materials and methods, isolates that produce PHA were isolated using enrichment media containing glucose from several liquid and solid samples taken from various locations in Egypt. A total of 156 isolates of bacteria were obtained and processed. After an initial screening that evaluated PHA production via the Nile red staining assay, six halophilic bacterial isolates exhibited significant fluorescence under UV light at a wavelength of 312 nm^46,47^. The isolates were subjected to an initial screening process using the Sudan Black B staining method to assess their PHB production^48^. Dark, blue-colored colonies that produced PHB were regarded as promising candidates for the synthesis of PHB. This suggests that the primary method by which these isolates might generate PHB from sucrose is chosen for additional quantitative screening. The six selected isolates were grown individually on a TYS medium enriched with 10% sodium chloride, glucose, and sucrose. The cultures were maintained at 37 °C for 72 h. The phenomenon of cellular proliferation was investigated. To extract PHB, the cells were subsequently collected, and evaluated, and the PHB content was compared. Table (4) reveals that two isolates, MH 46 and AD 1–2, generated PHB within the range of 0.57 –0.49 g/L, whereas four isolates produced PHB levels between 0.26 and 0.45 g/L. Cost-effective manufacturing necessitates the availability of inexpensive renewable sources for carbon feedstock and bacterial strains capable of generating substantial quantities of intracellular PHB utilizing these economical substrates. These wastes are abundantly accessible and provide substantial sources of carbohydrates generated by the agricultural industry. The six most potent halo-bacteria isolates selected were cultivated in a TYS medium containing 10% sodium chloride and glucose. Table (4) indicates that isolates ES 1–5 could not utilize orange peels as a carbon source, whereas isolate MH 46 exhibited superior production, attaining a peak PHB concentration of 1.008 g/L. The PHB production of the remaining isolates varied from 0.257 to 1.0 g/L. Ultimately, Table 4 exhibited the highest PHB production when orange peels served as carbon sources. Consequently, isolate MH 46 was employed as the most effective halo-bacterium for production and optimization, while orange peels served as a carbon source. Figure 1 shows Nile red staining of bacterial isolate MH 46 under UV light, and PHB after extraction.

**Table 4:** Quantitative primary screening test using glucose, sucrose, and orange peel, 6 halophilic bacterial isolates were chosen to produce PHB.

No.	Isolates code	PHB(g/L) ± SD from:		
		Glucose	Sucrose	Orange peels
26	SS 8 − 4	0.260 ± 0.003	0.258 ± 0.004	0.193 ± 0.007
46	MH 46	0.576 ± 0.007	0.546 ± 0.006	1.008 ± 0.018
47	AD 2	0.280 ± 0.037	0.243 ± 0.005	0.783 ± 0.007
66	ES 1–5	0.335 ± 0.021	0.306 ± 0.004	0
71	BW 4 − 2	0.455 ± 0.003	0.329 ± 0.004	0.257 ± 0.001
96	AD 1–2	0.490 ± 0.020	0.467 ± 0.004	1.000 ± 0.011

### Identification of the most potent bacterial isolate MH 46

#### Morphological and biochemical characteristics

**Fig. 2:**
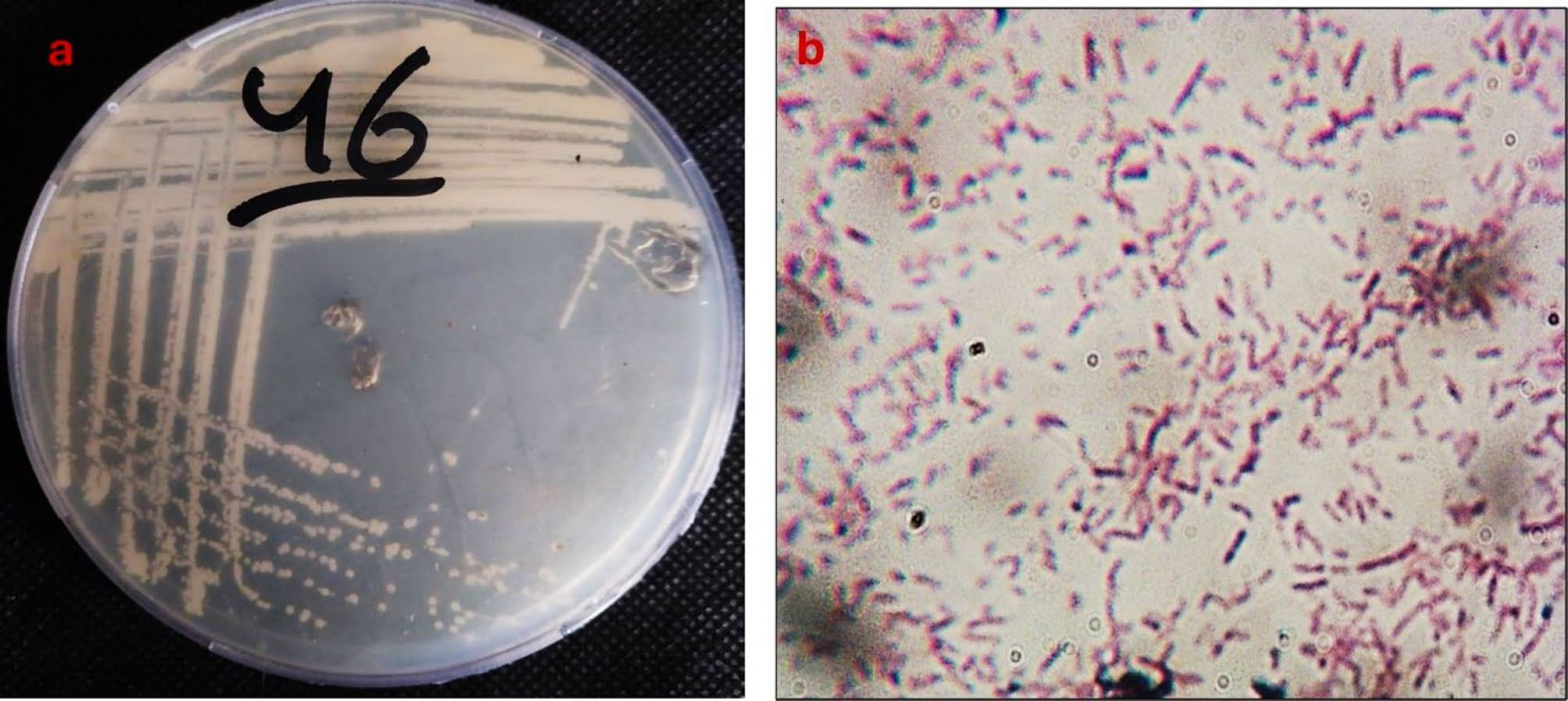
Shape of bacterial isolation MH 46 on an agar plate (**a**) and under the light microscope (**b**).

MH 46 was identified by examining the isolates’ morphology (Gram reaction, shape), biochemistry, and additional features. The strain MH 46 is rod-shaped, Gram-negative, has positive catalase activity (Fig. 2), and negative KOH reaction. It can flourish in conditions with up to 10% sodium chloride or elevated salinity.

#### Molecular identification and phylogenetic analysis

**Fig. 3:**
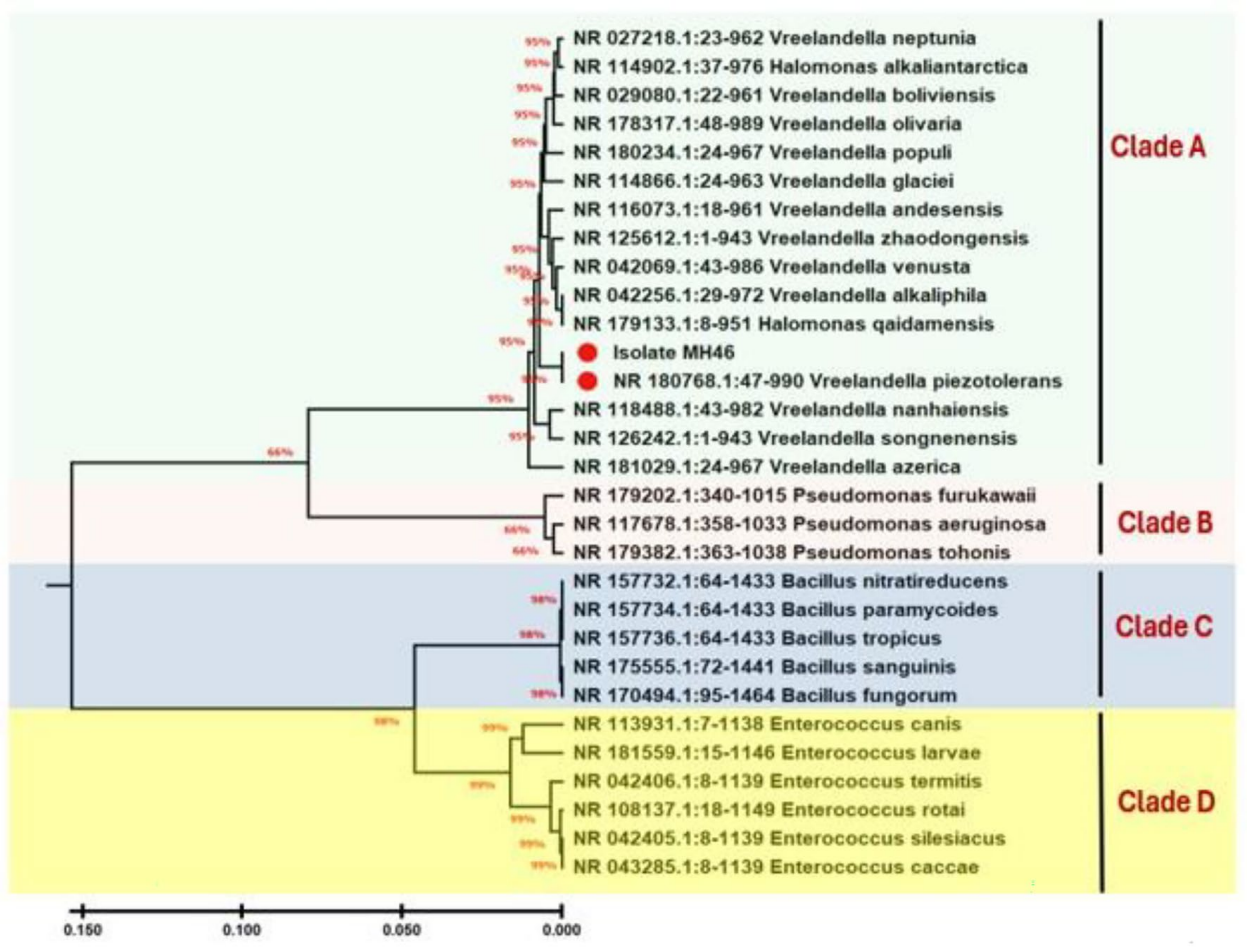
Phylogenetic tree derived from 16 S rRNA gene sequences illustrating the relative position of strain for *V. piezotolerans* MH 46. The tree was constructed using MEGA 11.

The 16 S rRNA gene of the bacterium MH46 was amplified and sequenced, enabling molecular identification. Following purification, sequencing, and alignment, blast analysis was used to compare the amplified PCR products to published sequences of 16 S rRNA gene strains kept in NCBI databases. The isolate with more than 99% similarity in its blast findings led to its identification as *Vreelandella piezotolerans*. The nucleotide sequences for this isolate have been cataloged in the GenBank database under accession number PP826284.1, the capacity of the genus *Halomonas* to synthesize PHA from various carbon sources is recognize^47,49^, (Fig. 3) shows their phylogenetic trees. The phylogenetic tree depicting isolates was created using MEGA11 (Fig. 3) to illustrate the evolutionary relationships among the clusters of these isolates. In the tree, Clade A, comprising isolate MH46, which were closely associated with *Vreelandella piezotolerans* NR 180768.1, exhibited a sequence similarity of 99.00%.

### Optimization of PHA using OFAT

**Fig. 4:**
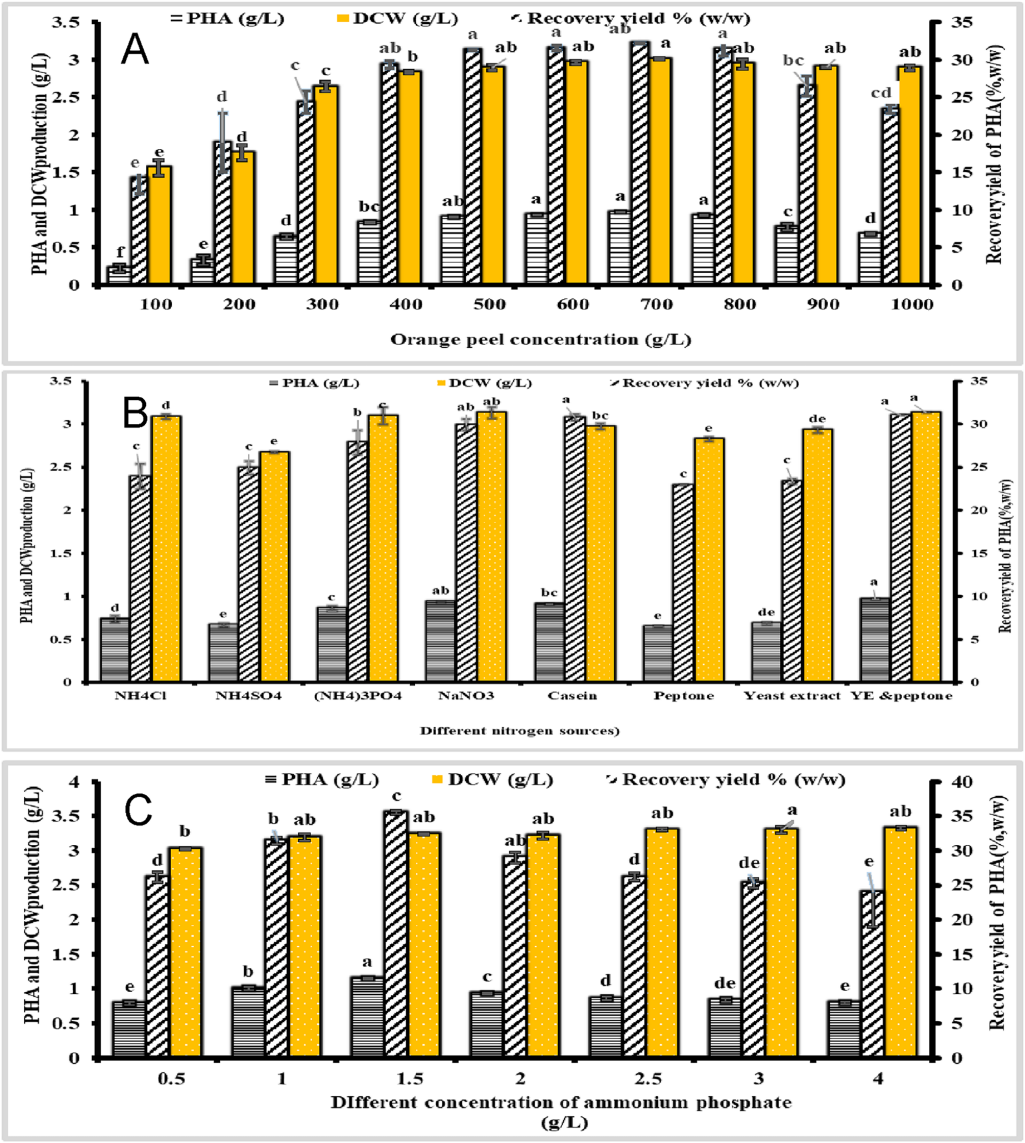
Influence of medium constituents on PHB production by strain MH 46. (**A**) Influence of orange peel concentration on PHB yield. (**B**) Influence of nitrogen source type on PHB yield. (**C**) Influence of peptone and yeast extract concentration on PHB yield. Each value is the mean of 3 replicates ± standard error of the mean. Different lower-case letters in the same bars are significantly different by post hoc-Tukey’s Honestly Significant Difference test (HSD) at *p* ≤ 0.05; values of the same bars with the same letters are not significantly different.

*Halomonas sp.* is capable of synthesizing polyhydroxyalkanoates (PHA) without the presence of nitrogen sources, magnesium ions, sulfate ions, or other components, provided that there is an adequate carbon supply in the environment^49,50^. This study utilized single-factor optimization experiments to investigate the effects of peptone and yeast extract concentrations (maintained at a 2:1 ratio), orange peel concentration, and the type of nitrogen source on the ability of strain MH 46 to produce polyhydroxybutyrate (PHB), and the PHB (Fig. 4), and the DCW and The PHB recovery yield is calculated using the formula (1). As illustrated in Fig. 4a, strain MH 46 was cultured in TYS medium at 37 °C with a pH of 7.0, using initial orange peel concentrations ranging from 100 to 1000 g/L. Results indicated that the dry cell weight (DCW) increased with orange peel concentrations of 1.56 g/L and 3.01 g/L, corresponding to overall concentrations of 100 g/L and 700 g/L, respectively. They showed significantly increased (p *<* 0.001) and an F-value of 44.63. However, when the concentration was raised from 700 g/L to 1000 g/L, a slight decrease in DCW was observed. The peak PHB production of 0.97 g/L showed a significant increase (p *<* 0.001) and an F-value of 235.71, and the highest PHB recovery yield of 32.23% (w/w) occurred at an orange peel concentration of 700 g/L showed a significant increase (p *<* 0.001), which was thus identified as the optimal concentration for efficient PHB fermentation. The influence of various organic and inorganic nitrogen sources on both growth and PHB production was assessed, as shown in Fig. 4b and c. Peptone and yeast extract yielded the best recovery, achieving 30.99% (w/w) showed a significant increase (p *<* 0.001) and an F-value of 53.60, although similar DCW (ranging from 2.67 to 3.13 g/L) was observed with other nitrogen sources, showed a significant increase (p *<* 0.001) and an F-value of 36.73. The maximum PHB synthesis from strain MH 46 reached 1.15 g/L showed a significant increase (p *<* 0.001) and an F-value of 137.25, with a recovery yield of 35.49% showed a significant increase (p *<* 0.001) and an F-value of 134.13. The optimal concentration for high-efficiency PHB production was determined to be 1.5 g/L.

Using single factor experiments, the first optimization phase examined the effects of orange peel concentration and peptone and yeast extract concentrations, which were kept at a 2:1 ratio, on strain MH 46’s ability to produce polyhydroxybutyrate (PHB). With a recovery yield of 32.23% (w/w) and a peak PHB production of 0.97 g/L, the results showed that an orange peel concentration of 700 g/L was ideal for fermentation. The best recovery was obtained with peptone and yeast extract at 30.99% (w/w), even though other nitrogen sources showed comparable dry cell weights. Additionally, DCW rose at 100 g/L and 700 g/L orange peel concentrations but decreased at 1000 g/L, according to the study. Maximum PHB synthesis was found to be 1.15 g/L, and the ideal concentration for effective production was assessed at 1.5 g/L.

**Fig. 5:**
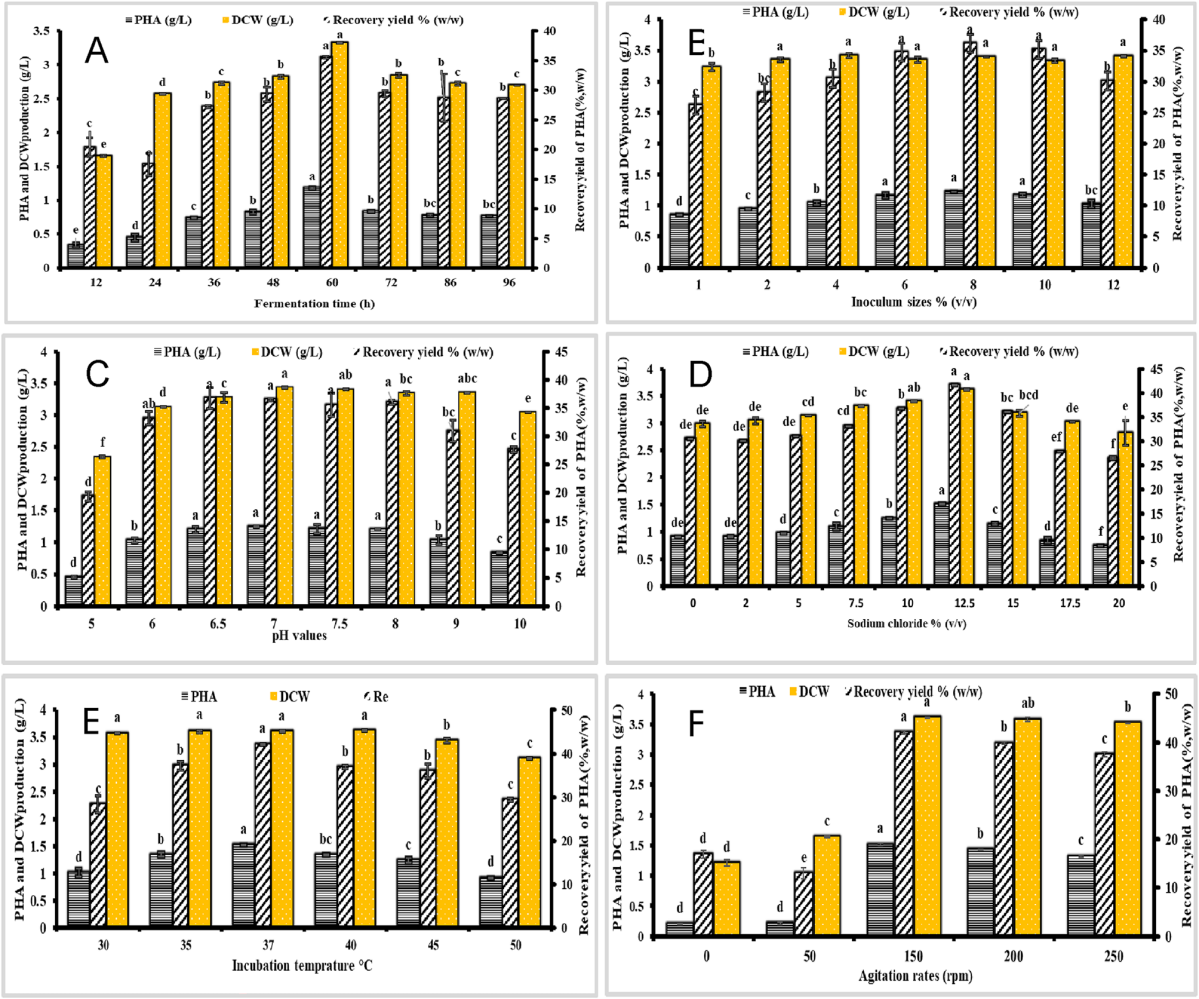
Influence of factors on PHB synthesis by strain MH 46. (**a**) Influence of fermentation time on PHB production. (**b**) Influence of inoculum size on PHB production. (**c**) Influence of pH on PHB production. (**d**) Influence of sodium chloride on PHB yield. (**e**) Influence of temperature on PHB production. (**f**) Influence of agitation rate and inoculation size on PHB production. Each value is the mean of 3 replicates ± standard error of the mean. Different lower-case letters in the same bars are significantly different by post hoc-Tukey’s Honestly Significant Difference test (HSD) at *p* ≤ 0.05; values of the same bars with the same letters are not significantly different.

To further analyze the impact of fermentation conditions on PHB production by strain MH 46, experiments were conducted to optimize fermentation duration, inoculum size, pH, sodium chloride concentration, temperature, and agitation rate. The results are presented in Fig. 5. Strains were cultured for varying durations (12 to 96 h) at 150 rpm and 37 °C, as shown in Fig. 5A. The accumulation of growth-associated PHB in strain MH 46 was noted during the exponential growth phase (Fig. 5a). The maximum PHB production of 1.18 g/L showed a significant increase (p *<* 0.001) and an F-value of 344.59 and peak DCW of 3.32 g/L showed a significant increase (p *<* 0.001) and an F-value of 1704.88 occurred at 60 h, with a maximum recovery yield of 35.54% (w/w) a significant increase (p *<* 0.001) and an F-value of 92.32. Extending the incubation period to 96 h resulted in a decline in both DCW (to 2.69 g/L) and PHB production (to 0.768 g/L).

As indicated in Fig. 5b, a correlation was observed between increasing DCW and PHB generation as inoculum size concentration increased for strain MH 46. The highest DCW of 3.4 g/L showed a significant increase (p *<* 0.001) and an F-value of 9.81 and PHB production of 1.23 g/L showed a significant increase (p *<* 0.001) and an F-value of 66.12 were recorded at 60 h, with a maximum recovery yield of 36.18% (w/w) showed a significant increase (p *<* 0.001) and an F-value of 52.93. PHB accumulation and recovery yield decreased slightly at inoculum concentrations above and below this threshold, with the optimal yield achieved at 8% inoculum size.

Figure 5c illustrates that DCW remained relatively stable across a broad pH range (6.0 to 10.0), varying from 3.04 to 3.40 g/L after 60 h showed a significant increase (p *<* 0.001) and an F-value of 455.32. At an initial pH of 5, DCW dropped to 2.34 g/L, indicating a significant decline. However, PHB production and recovery yield increased significantly with a rise in initial pH, peaking at pH 7, where 1.25 g/L showed a significant increase (p *<* 0.001) and an F-value of 122.56 and 36.44% (w/w) showed a significant increase (p *<* 0.001) and an F-value of 61.95 were recorded. This result is consistent with the findings of Abdel-Rahman, Desouky^51^, which found that the DCW is nearly identical across a broad pH range (6.0–8.0), with a range of 3.41–5.27 g/L after 72 h that is comparable to the value obtained after 48 h (3.01–5.26 g/L). However, at pH 6.5, the maximum PHB production and recovery yield were 2.36 g/L and 44.78% (w/w), respectively.

In Fig. 5d, it is shown that strain MH 46 produced maximum DCW (3.62 g/L) showed a significant increase (p *<* 0.001) and an F-value of 26.93 and PHB (1.51 g/L) showed a significant increase (p *<* 0.001) and an F-value of 238.65 at a sodium chloride concentration of 12.5% after 60 h post-inoculation. This condition also yielded a maximum recovery of 41.71% (w/w) showed a significant increase (p *<* 0.001) and an F-value of 51.64. Similarly, PHB accumulation and recovery yield slightly decreased at sodium chloride concentrations below and above this optimal threshold.

As presented in Fig. 5e, strain MH 46 achieved a DCW of 3.61 g/L showed a significant increase (p *<* 0.001) and an F-value of 277.37 after 60 h at 37 °C, resulting in the highest PHB recovery yield of 42.11% (w/w) showed a significant increase (p *<* 0.001) and an F-value of 65.75 and PHB production of 1.52 g/L showed a significant increase (p *<* 0.001) and an F-value of 110.81. Therefore, the optimal incubation temperature for high-efficiency PHB production was established. This result is consistent with Desouky, Abdel-Rahman^27^ and Abdel-Rahman, Desouky^51^, which reported that all optimized agents were prepared at 37 °C. Finally, as depicted in Fig. 5f, after 60 h at an agitation rate of 150 rpm, the highest DCW of 3.61 g/L showed a significant increase (p *<* 0.001) and an F-value of 5117.71 and PHB concentration of 1.52 g/L showed a significant increase (p *<* 0.001) and an F-value of 7315.01 were obtained. This result is consistent with the findings of Desouky, Abdel-Rahman^27^, which highlight the importance of stirring for effective mixing and mass and heat transfer. The effect of varying agitation rates (0.0 [fixed], 50, 150, 200, and 250 rpm) on the growth and PHB production of strain AZU-A2 was investigated. The highest dry cell weight (DCW) of 4.39 g/L and the maximum PHB concentration of 3.0 g/L were observed after 24 h at an agitation rate of 200 rpm.

To improve the fermentation conditions for strain MH 46’s production of PHB, the second optimization phase evaluated variables like temperature, agitation rate, pH, sodium chloride concentration, and inoculum size. With a recovery yield of 30.58% (w/w), the results showed that the highest PHB production (0.902 g/L) and peak dry cell weight (DCW) of 2.95 g/L happened at 60 h of fermentation. With the highest DCW (3.4 g/L) and PHB production (1.23 g/L) at this duration, an ideal inoculum size of 8% was identified. PHB production peaked at a starting pH of 7 and a sodium chloride concentration of 12.5%, according to the study. According to earlier research, 37 °C was also found to be the ideal incubation temperature for high-efficiency PHB production.

The two optimization stages show a thorough method for increasing PHB yield by adjusting fermentation conditions and optimizing nutrients. The experimental design of the second OFAT was influenced by the findings of the first, enabling a more focused examination of the circumstances that optimize PHB production in strain MH 46.

### Results of response surface optimization utilizing Plackett–Burman design (PBD) and Box-Behnken design (BBD)

Traditional optimization methods are often time-consuming, prone to errors, and do not facilitate the simultaneous analysis of multiple interacting factors^50^; however, Data-driven treatment approaches can be created through statistical design. The yield of the product can be raised by optimizing the fermentation process. Numerous applications have been made for biological process statistical optimization using PBD and RSM^52^. According to reports, PHA yield optimization can be accomplished using the RSM optimization strategy^50,53^. To identify the ideal fermentation conditions, this study used PBD and BBD in multi-factor interaction tests.

#### Results of PBD experiments

Fifteen trials were conducted to examine eleven factors of media components; trial number eleven produced the most PHB (Table 5), while trial number four produced the least (Table 6 includes the regression coefficients and confidence levels). The media components demonstrated both favorable and unfavorable impacts on PHB yield. As illustrated in Fig. 4, statistical analysis (t values) revealed that the most effective factors were agitation rate (F), inoculum size (B), and fermentation temperature (E). Table 4 presents the ANOVA results for the Plackett–Burman design concerning PHB production, where the first-order model’s determinant coefficient (R^2^) for PHB production was 0.9987. The model’s 9183.46 F-value indicates that it is significant. A significant “Model F-value” like this is 0.01% likely to result from noise. Additionally, the curvature F-value of 37818.90 implies substantial curvature in the design space, as assessed by the difference between the averages of the center and factorial points, with a similar 0.01% likelihood of being attributed to noise.

Stepwise regression analysis was conducted using Design Expert 7.0, leading to the formulation of the following predictive equation for PHB yield (Y):

4 $${\text{Y}}\,=\,{\text{12}}.{\text{93}}--0.{\text{1484A}}\, - \,0.{\text{8568B}}\, - \,0.{\text{7612C}}\, - \,{\text{1}}.{\text{85D}}\, - \,{\text{4}}.{\text{81E}}\,+\,{\text{1}}.{\text{42F}}$$

Finally, as indicated in Table 5 of Round 11, the maximum PHB concentration production following PBD was 2.144 g/L, whereas following OFAT optimization, we achieved a PHB concentration production of 1.52 g/L. Understanding complex biological systems where joint effects can be significant requires the ability to explore potential interactions between variables, which PBD analysis facilitates in contrast to OFAT analysis, which may overlook such interactions. OFAT typically examines one variable at a time, potentially overlooking significant interactions that could enhance yield.

**Table 5:** Summarizes the Plackett–Burman experimental design utilized for screening the culture conditions affecting PHB production.

Run	A	B	C	D	E	F	G	H	J	K	L	PHB (g/L)
1	− 1	1	− 1	1	1	− 1	1	1	1	− 1	− 1	1.797
2	− 1	1	1	− 1	1	1	1	− 1	− 1	− 1	1	1.682
3	1	− 1	-1	− 1	1	− 1	1	1	− 1	1	1	1.075
4	1	1	1	− 1	− 1	− 1	1	− 1	1	1	− 1	0.282
5	− 1	− 1	1	− 1	1	1	− 1	1	1	1	− 1	1.355
6	− 1	− 1	− 1	1	− 1	1	1	− 1	1	1	1	0.66
7	0	0	0	0	0	0	0	0	0	0	0	1.89
8	1	− 1	1	1	1	− 1	− 1	− 1	1	− 1	1	0.747
9	− 1	− 1	− 1	− 1	− 1	− 1	− 1	− 1	− 1	− 1	− 1	1.391
10	0	0	0	0	0	0	0	0	0	0	0	1.9
11	1	1	− 1	1	1	1	− 1	− 1	− 1	1	− 1	2.144
12	0	0	0	0	0	0	0	0	0	0	0	1.9
13	− 1	1	1	1	− 1	− 1	− 1	1	− 1	1	1	0.563
14	1	− 1	1	1	− 1	1	1	1	− 1	− 1	− 1	0.99
15	1	1	− 1	− 1	− 1	1	− 1	1	1	− 1	1	1.377

A, MgCl_2_.6H_2_O (g/L); B, Temperature (^o^C); C, Sodium chloride (%); D, Incubation time (hr.); E, Inoculum size (%) (v/v); F, Agitation rate (rpm); G, Yeast extract (g/L); H, MgSO_4_.7H_2_O (g/L); J, Orange peel concentration (g/L); K, Peptone (g/L); L, Initial pH.

**Table 6:** Provides the results of the main effect analysis for each factor in the Plackett–Burman experimental design.

Source	Sum of squares	Df	Mean square	F value	*P* value
Model	3.37	11	0.31	9183.46	0.0001
MgCl_2_.6H_2_O	0.058	1	0.058	1734.72	0.0006
Temperature	0.22	1	0.22	6617.82	0.0002
Sodium chloride	0.67	1	0.67	19951.56	< 0.0001
Incubation time	5.677E-003	1	5.677E-003	170.30	0.0058
Inoculum size	1.04	1	1.04	31275.92	< 0.0001
Agitation rate	0.46	1	0.46	13841.52	< 0.0001
Yeast extract	0.099	1	0.099	2975.70	0.0003
MgSO_4_.7H_2_O	5.250E-003	1	5.250E-003	157.50	0.0063
Orange peel concentration	0.22	1	0.22	6617.82	0.0002
Peptone	0.30	1	0.30	9072.56	0.0001
pH	0.29	1	0.29	8602.56	0.0001
Curvature	1.26	1	1.26	37818.90	< 0.0001
Pure Error	6.667E-005	2	3.333E-005		
Cor Total	4.63	14			

Detemination cofficient R^2^ = 1; Adjusted determination R^2^ = 0.9999; Coefficient of variation CV = 0.44%

**Fig. 6:**
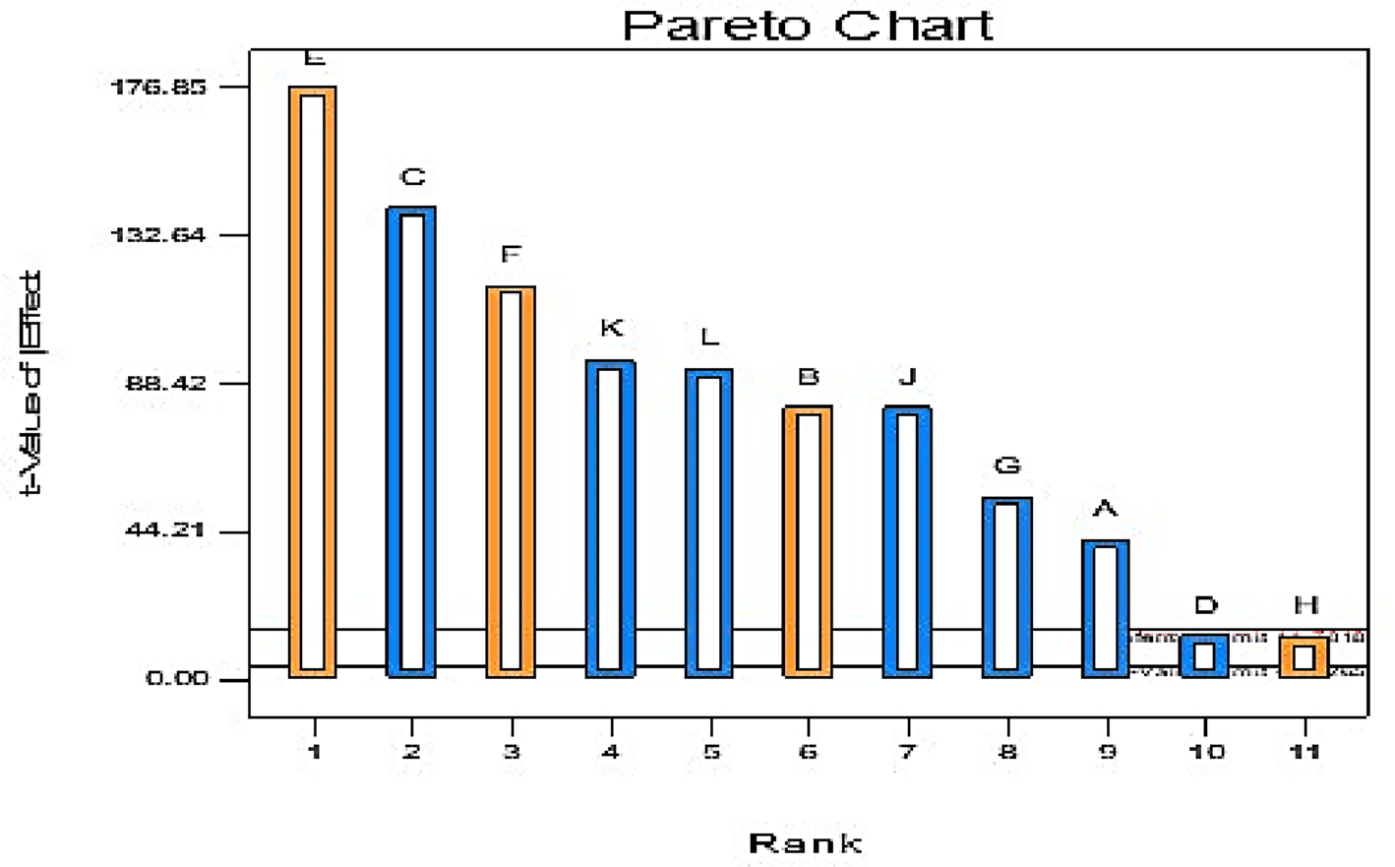
The Pareto chart shows significant factors affecting PHB production by strain MH 46.

#### Response surface methodology

According to the results above, PHB production was greatly impacted by three important factors: inoculum size, agitation rate, and fermentation temperature. These factors were further analyzed using Box-Behnken Design (BBD), as detailed in Table 7. The resulting second-order polynomial equation is expressed as:

5 $${\text{Y}}\,=\,+\,{\text{2}}.{\text{14}}--0.0{\text{33A}}\,+\,0.{\text{24B }} - \,0.{\text{13C }} - \,0.0{\text{4}}0{\text{AB}}\,+\,0.{\text{14AC }} - \,0.0{\text{99BC}}\,+\,0.{\text{47A2}}\,+\,0.{\text{49B2}}\,+\,0.{\text{74C2}}$$

Where A, B, and C represent the concentrations of inoculum size, agitation rate, and fermentation temperature, respectively, while Y corresponds to PHB production (g/L). The coefficients indicate the influence of linear, interactive, and quadratic terms on PHB yield. ANOVA analysis alongside F-tests was employed to evaluate the statistical significance of the model (Table 8).

The model demonstrated a high degree of reliability, with an R² value of 0.9522, suggesting that it accounts for 95.22% of the variability in the response (Fig. 6). The significance of the model is supported by an F-value of 15.48, with only a 0.08% likelihood that such an F-value could arise from noise. Model terms with “Prob > F” values below 0.0500, including B, A², B², and C², were identified as significant.

For strain, MH 46, which utilizes orange peel as a carbon source for PHB synthesis, the optimal parameters identified through Design Expert 7.0 software were an inoculum size of 10%, an agitation rate of 180 rpm, and a temperature of 38.8 °C (Fig. 6).

**Table 7:** Experimental design and results of Box–Behnken optimization experiment.

Trials	A (inoculum size)		B (agitation rate)		C (incubation temp.)		PHB (g/L)
	Coded level	Real level (%)	Coded level	Observed	Coded level	Real level (^o^c)	Observed
1	− 1	6	0	150	1	40	3.236
2	0	8	1	180	− 1	35	3.689
3	1	10	− 1	120	0	37	2.846
4	0	8	0	150	0	37	2.144
5	0	8	− 1	120	1	40	3.249
6	0	8	1	180	1	40	3.249
7	− 1	6	− 1	120	0	37	2.597
8	0	8	0	150	0	37	2.144
9	0	8	0	150	0	37	2.144
10	0	8	0	150	0	37	2.144
11	0	8	0	150	0	37	2.144
12	1	10	0	150	− 1	35	3.19
13	− 1	6	1	180	0	37	3.44
14	1	10	0	150	1	40	3.218
15	− 1	6	0	150	− 1	35	3.78
16	1	10	1	180	0	37	3.531
17	0	8	-1	120	-1	35	3.294

A, inoculum size (%); B, agitation rate (g/L) and C, incubation temperature (h).

**Table 8:** ANOVA results for response surface.

Source	Sum of squares	Df	Mean square	F value	*P*- Value
Model	5.431	9	0.603	15.48	0.0008
A-Inoculum Size	0.009	1	0.009	0.23	0.00646
B-Agitation rate	0.462	1	0.462	11.86	0.0108
C-Temperature	0.125	1	0.125	3.21	0.0116
AB	0.006	1	0.006	0.16	0.0070
AC	0.082	1	0.082	2.10	0.0190
BC	0.039	1	0.039	1.00	0.0350
A^2^	0.941	1	0.941	24.12	0.0017
B^2^	0.998	1	0.998	25.60	0.0015
C^2^	2.302	1	2.302	59.04	0.0001
Residual	0.273	7	0.039		
Lack of Fit	0.273	3	0.091		
Pure Error	0.000	4	0.000		
Cor Total	5.704	16			

Determination coefficient R^2^ = 0.9522; Adjusted determination R^2^ = 0.8906; Coefficient of variation CV = 6.71%; model is significant.

#### Experimental verification based on the optimization results

**Fig. 7:**
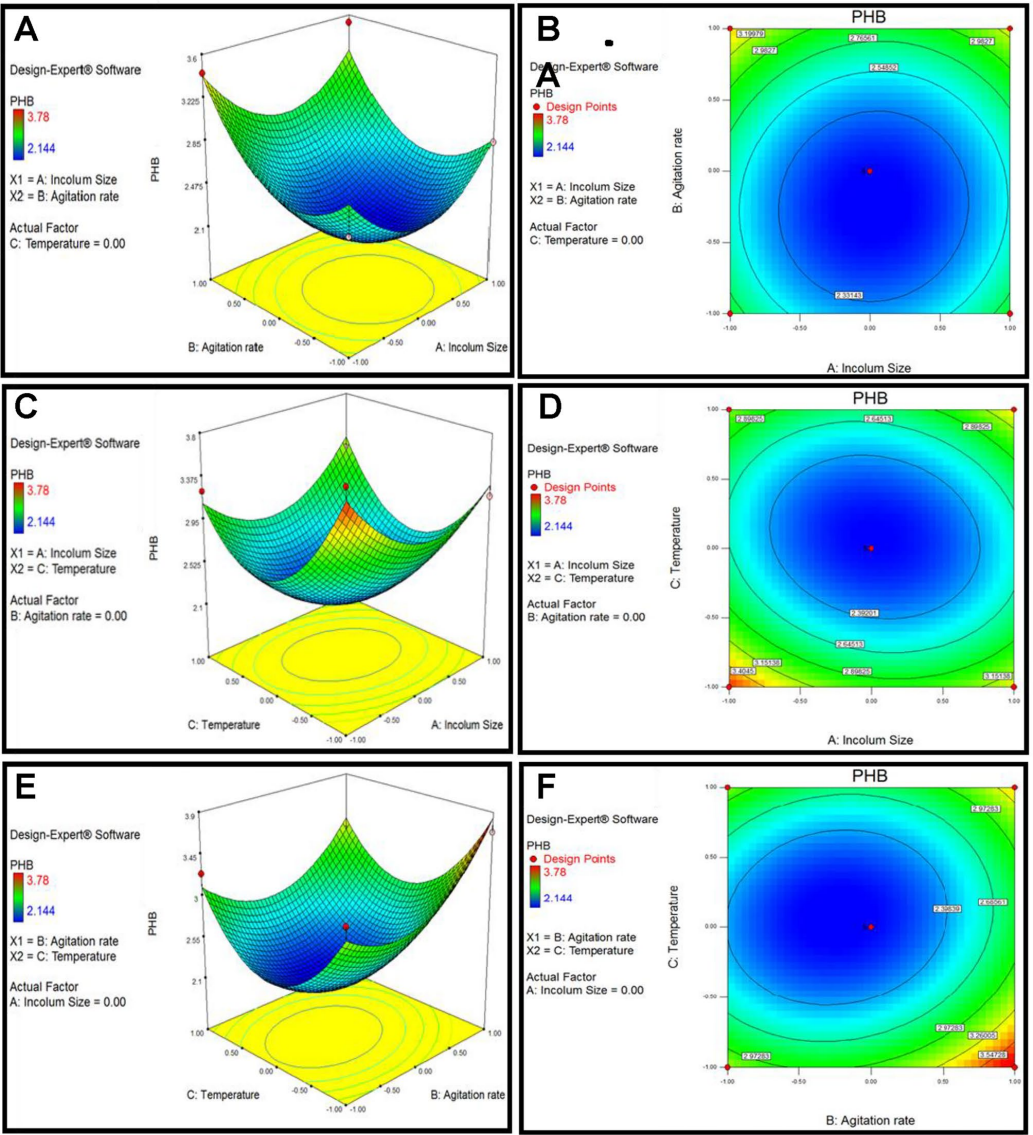
Response surface plots of three variables in medium on PHB production. (**A**,**B**) Interaction of agitation rate and inoculum size; (**C**,**D**) interaction of temperature and inoculum size; (E&F) interaction of temperature and agitation rate.

The response surface indicated that the ideal parameters were temperature 38.8 °C, agitation rate 180 rpm, and inoculum size 10. The yield of PHB was 3.912 g/L. Three fermentation trials were conducted under these parameters. After 72 h, the PHB yield was measured, confirming the model’s significance in predicting PHB synthesis and its capability to represent the effects of various factors on yield. The strong relationship between the predicted and experimental results attests to the quadratic model’s dependability in estimating the combined effects of several variables on PHB biosynthesis. The convex shape of the 3D response surface plot highlighted how inoculum size concentrations, agitation rate, and fermentation temperature interact to maximize PHB yield. The observed patterns indicate that these parameters significantly impact microbial metabolism, nutrient assimilation, and polymer accumulation. The strong relationship between the model’s predictions and actual results demonstrates how well the RSM model optimizes PHB production. These results support the model’s predictive accuracy and offer a foundation for scaling up PHB biosynthesis using renewable and inexpensive feedstocks. Response surface plots of three variables in medium on PHB production are shown in Fig. 7.To assess the interactions among various parameters and identify their optimal levels for enhanced PHB synthesis, the effects of inoculum size (A), agitation speed (B), and temperature (C) were illustrated in Fig. 7. In particular, Fig. 7A, B demonstrates the combined influence of inoculum size and agitation on PHB yield. An increase in inoculum size from 6.0 to 10% resulted in a notable improvement in PHB production. However, when the inoculum size dropped below 6.0%, a decline in PHB accumulation was observed. Similarly, raising the agitation speed from 120 to 180 rpm led to an increase in PHB synthesis. The influence of inoculum size (A) and temperature (C) on PHB production is illustrated in Fig. 7C and D. A reduction in temperature from 40 °C to 35 °C led to an increase in PHB yield. Additionally, a higher inoculum size resulted in a significant enhancement in PHB production. Figure 7E and F present the effects of agitation and temperature during cultivation. Here again, PHB accumulation improved as the temperature decreased from 40 °C to 35 °C, while temperatures exceeding 40 °C caused a sharp decline in PHB synthesis.

The significance of employing a response surface methodology (RSM) to enhance yield has been highlighted by^50^. Their study identified the optimal conditions for polyhydroxybutyrate (PHB) production as follows: an initial pH of 6.7, a temperature of 25 °C, and a culture volume of 124 mL. Under these conditions, the maximum PHB yield reached 28.8% of the dry cell weight (DCW). To validate these findings, the fermentation process was repeated three times using the optimized parameters, and PHB content was measured after 24 h. The average yield was 28.1%, representing a 35.2% improvement compared to the yield prior to optimization. Chakraborty et al. (2012)^54^ investigated polyhydroxyalkanoate (PHA) production in *Ralstonia eutropha* cultivated with intermittent feeding of short-chain fatty acids. The inoculum was prepared to a mean optical density of 1.04 at 600 nm (OD_600_), and the culture was maintained in a cost-effective medium known as CCS (solubilized corn substrate) under controlled conditions (30 °C, 250 rpm) for 24 h. Notably, when artificial rumen fluid (ARF) was administered at three-hour intervals over a 61-hour fermentation period, the process yielded a high PHA concentration (8.37 g·L^−1^), a PHA content of 39.52%, and a productivity rate of 0.0697 g·L^−1^·h^−1^. Despite some variability, the productivity reached 0.19 g·L^−1^·h^−1^ within the first 24 h, a result that closely aligns with the findings reported in the current study.

Factorial design has become a widely adopted and valuable methodological approach for optimizing process conditions. Unlike traditional one-variable-at-a-time methods, factorial design allows for the simultaneous evaluation of multiple factors, thereby offering greater efficiency and insight into variable interactions. Numerous studies have employed this technique to enhance culture conditions for microbial processes^55^. More variables or different media components could be included in future research to improve PHB production even more. This could entail evaluating various carbon sources or nutrient compositions that might work in concert with the important factors that have been identified.

### Characterization of polyhydroxyalkanoates

#### GC-MS analysis

**Fig. 8:**
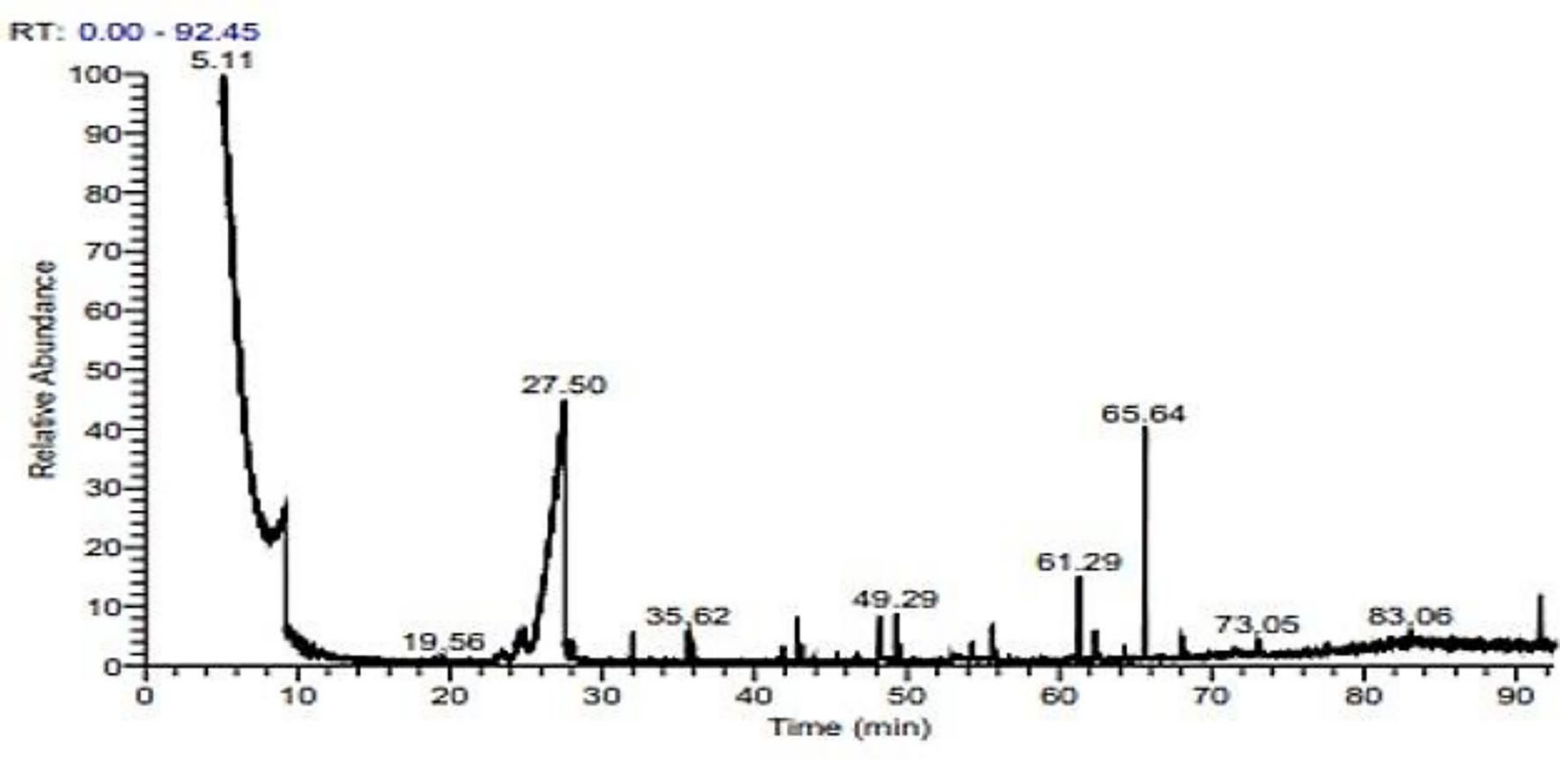
GC-MS spectral chromatogram of derivatives PHB obtained from *Vreelandella piezotolerans*.

The molecular structure of PHB may be quantified and characterized very well using gas chromatography. For GC analysis, PHB must be depolymerized into acids, diols, or esters^56^. After the PHB sample underwent methanolysis, the methyl esters showed fragmentation patterns in GCMS, which made it possible to identify the PHB derivatives that were formed. By *Vreelandella piezotolerans* (Fig. 8) four major peaks were identified in the extract of the biopolymer, The retention times for these peaks were as follows: 21.13, 23.25, 24.72, and 46.75 min. Dimers of ç-hydroxy butyric acid, crotyl ester, and ç-hydroxybutyrate are among them, as are 2-butenoic acid, 1-methyl ethyl ester, and hexadecanoic acid, methyl ester. Table 9 shows the principal peaks and supports the presence of polyhydroxy butyric acid (PHB) in *V. piezotolerans* extracts. This result matched with^57^.

**Table 9:**
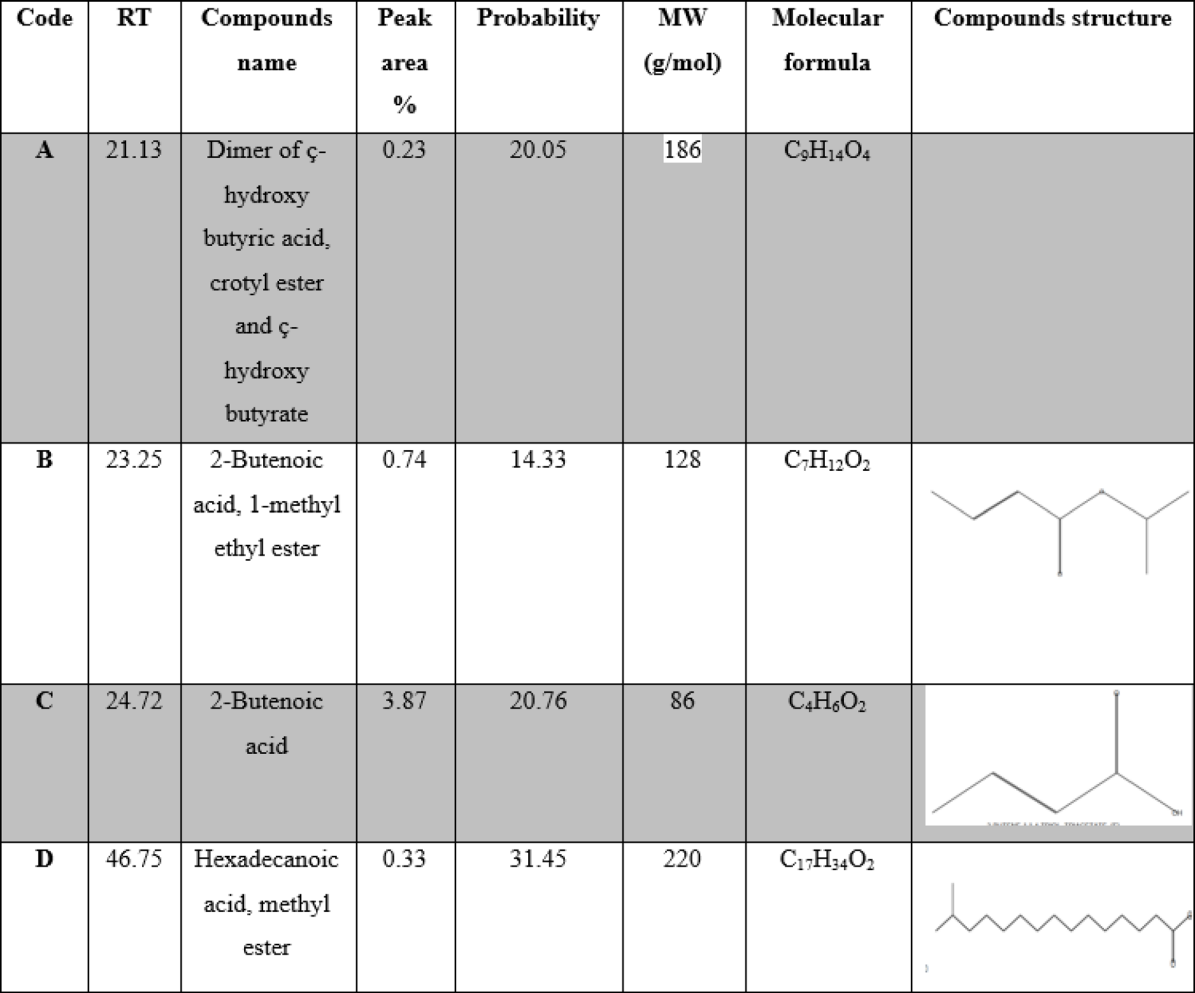
List of PHB derivatives detected in GC-MS

#### Fourier transform-infrared spectroscopy (FTIR)

**Fig. 9:**
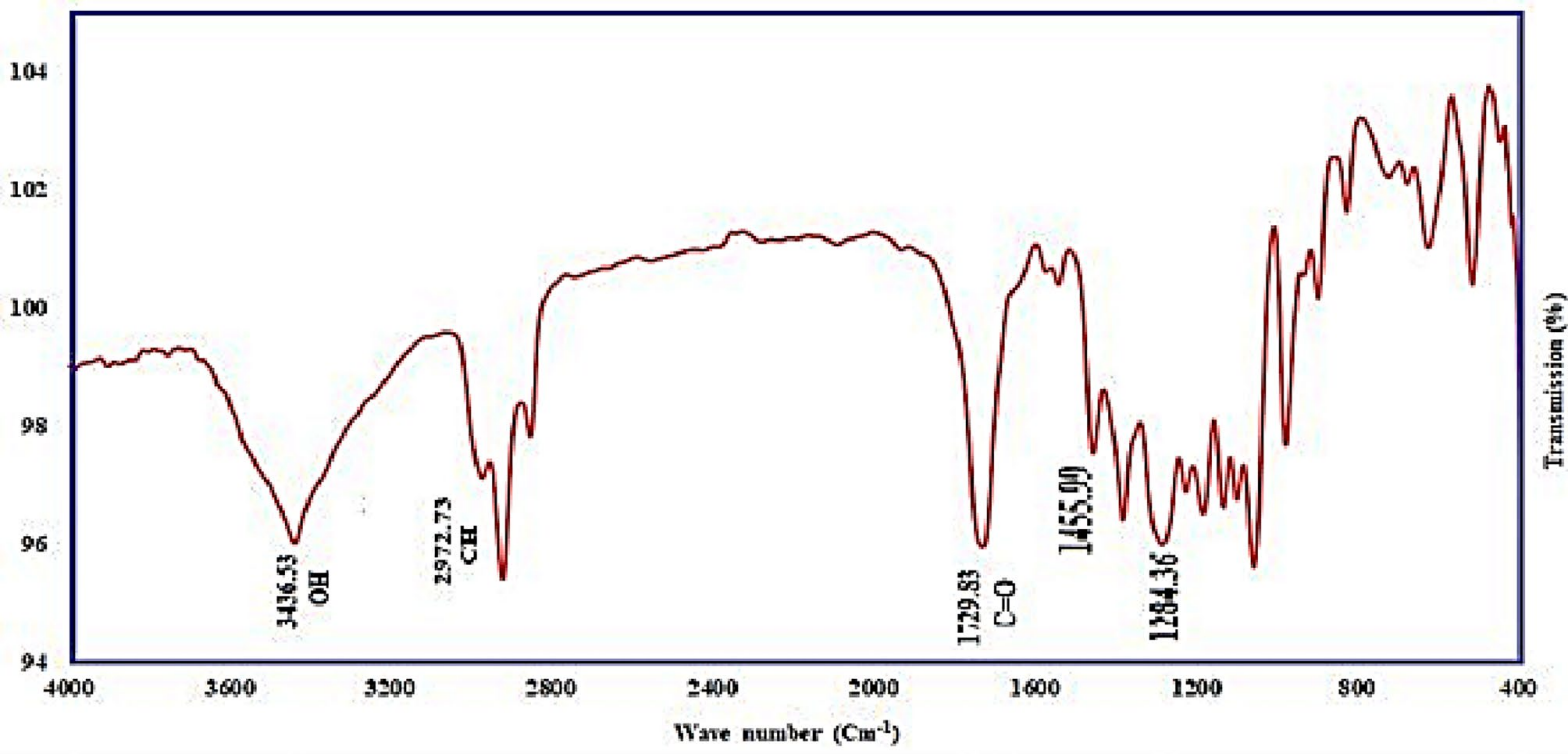
Fourier transform infrared spectra analysis of PHB synthesized by *Vreelandella piezotoleran.*

The FTIR analysis of the PHA produced by *V. piezotolerans* revealed several significant absorption bands. Specifically, absorption bands were observed at 3436.53 cm⁻¹ (indicative of OH stretching), 2972.73 cm^−1^ (C-H stretching), 1729.83 cm^−1^ (C = O stretching), and at 1455.99 and 1284.36 cm^-1^ (methylene bending) (Fig. 9). These findings are in agreement with previous studies^27^, which reported absorption bands at 3453 cm⁻¹ (OH stretching), 2980 cm^−1^ (C-H stretching), 1448 cm^-1^, and 1285 cm^-1^ (methylene bending), as well as 1736 cm⁻¹ for C = O stretching. Furthermore, these results align with the recent research by^58^, which highlighted the C = O characteristic band as a key feature for PHAs isolated from *Haloferax mediterranei*, detected within the 1720–1740 cm⁻¹ range. Additional findings from^59^ Described a PHA derived from *B. endophyticus*, characterized by a distinctive C = O stretch at 1726 cm^−1^ Moreover, strong adsorption bands observed around 1379, 1458, 2929, 1649, and 3749 cm⁻¹ correspond to the functional groups –CH_2_, –CH_2_, CH, C–O, and O–H, respectively, which are characteristic of pure PHB.

#### Nuclear magnetic resonance (NMR)

**Fig. 10:**
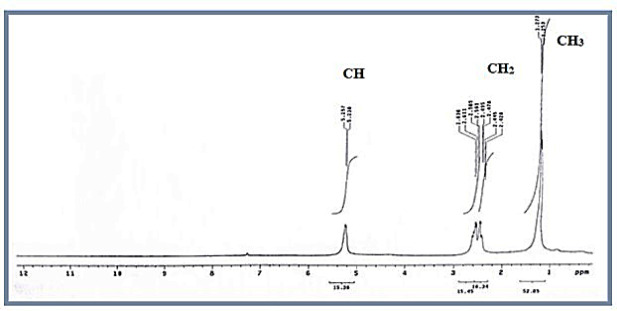
^1^H-NMR spectra of PHB produced by *Vreelandella piezotolerans*.

**Fig. 11:**
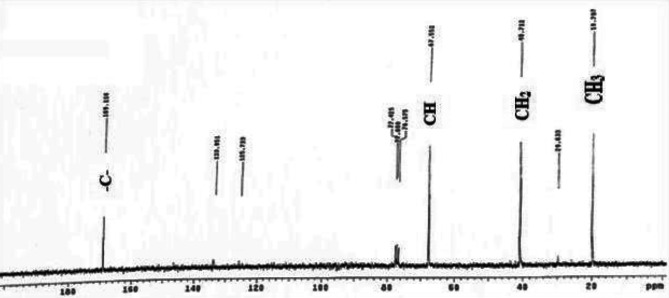
^13^C-NMR spectra of PHB produced by *V. piezotolerans*.

The ¹H-NMR spectrum of the PHB extracted from strain MH 46 (Fig. 10) displayed characteristic signals consistent with the structure of PHB. A doublet at 1.253 ppm was attributed to the methyl (–CH₃) group, coupled to one proton, while a doublet of quadruplet at 2.445 ppm corresponded to the methylene (–CH₂) group adjacent to an asymmetric carbon atom with a single proton. Additionally, a signal at 5.238 ppm was ascribed to the methine (–CH) group. The observed chemical shifts and assignments correlated well with an authentic PHB sample from Aldrich, confirming the extracted biopolymer as poly-3-hydroxybutyric acid, further supported by the ¹³C NMR spectrum. These findings are consistent with the data presented by^51^, which identified characteristic signals of PHA. Specifically, a doublet at 1.25 ppm was attributed to the methyl (–CH₃) group, which is coupled to one proton, while a doublet of quadruplet at 2.44 ppm corresponded to the methylene (–CH_2_) group adjacent to an asymmetric carbon atom bearing a single proton. Additionally, a signal at 5.21 ppm was ascribed to the methine (–CH) group. This data aligns with previously published results for the same compound^27,60^. Furthermore, the ¹³C NMR spectra (Fig. 11) exhibited peaks at 19.707, 40.712, 67.552, and 169.116 ppm, corresponding to the resonances for (–CH₃), (–CH₂–), (–CH–), and (–C–), respectively. The polymer produced was confirmed to be PHB through the resonances of the methyl, methylene, methine, ester groups, and carbonyl carbon atoms. This data corroborates with the ¹³C NMR spectra, which indicated peaks at 19.71, 40.75, 67.63, and 169.43 ppm for (–CH₃), (–CH₂), (–CH), and (–C)^57^.

#### X-ray diffraction

The XRD analysis of the polymer extracted from *V. piezotolerans* revealed a distinct crystallization pattern characteristic of PHB. The XRD profile displayed prominent diffraction peaks at 2θ values of 13.547°, 16.009°, 16.428°, 20.198°, 20.382°, 27.933°, 29.654°, 30.603°, and 31.590° (Table 10). These results align with findings reported by^50^, which identified similar peaks in the XRD results for strain L17, corresponding to the (020), (110), (101), (121), and (002) planes, with peak positions at 13.6°, 17.24°, 21.4°, 25.7°, and 30.5°. Additionally, the values are comparable to those presented in^44^, which reported the XRD pattern of a polymer derived from glucose and standard PHB, noting distinctive peaks at 2θ values of 13, 17, 20, 21, 22, and 25. The 2θ values for standard PHB (13.55°, 17.05°, 20.04°, 21.61°, 22.55°, and 25.59°) are also similar to those observed in this study. The orange peel polymers’ XRD pattern demonstrated strong crystalline and resembled the standard PHB well.

**Table 10:** XRD graph represents the crystalline characteristic of PHB for the isolate *V. piezotolerans*.

Angle	d Value	Net Intensity	Rel. Intensity	FWHM
13.547 °	6.53103 Å	96.6563	35.0%	0.214
16.009 °	5.53160 Å	222.894	80.8%	0.809
16.428	5.39144 Å	231.346	83.8%	0.809
20.198 °	4.39295 Å	140.467	50.9%	0.537
20.382 °	4.35372 Å	116.125	42.1%	0.537
27.933 °	3.19158 Å	204.940	74.3%	0.100
29.654 °	3.01018 Å	166.460	60.3%	0.100
30.603 °	2.91889 Å	275.980	100%	0.100
31.590 °	2.82997 Å	241.178	87.4%	0.100

#### Thermal characterization of the purified polymer

**Fig. 12:**
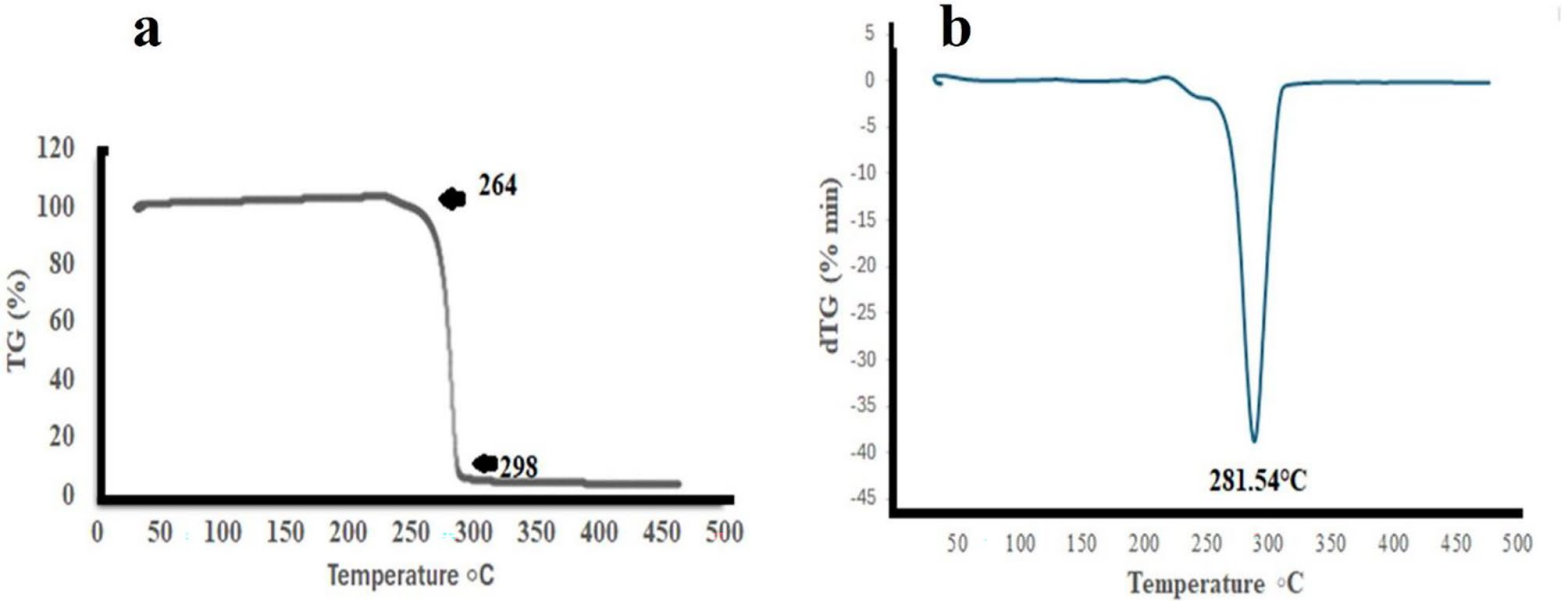
Thermal analysis of *V. piezotolerans* PHB by TGA.

TGA studies are crucial for determining the processing limits of thermoplastic materials, particularly for PHB, which has a narrow processing window^61^. The TGA analysis revealed a two-step decomposition process for the sample, with an initial thermogravimetric loss occurring between 30 and 100 °C, attributed to the loss of water. Adequate drying was conducted before TGA detection to mitigate any impact from this initial decomposition phase. The second decomposition phase commenced at 264 °C, with complete degradation of PHB observed at 298 °C (Fig. 12a). At this temperature, the ester bonds were broken, resulting in shorter chain fragments containing carboxylic acid and olefinic terminal groups^62^. The decomposition temperature (Td) for PHB produced by *V. piezotolerans* was determined to be 298 °C. This finding is supported by the DTG curve, which indicated a peak weight loss rate at this temperature, suggesting that the PHB produced is of good quality (Fig. 12b). Moreover, these results align closely with reference samples from prior research (Huang et al., 2023).

**Fig. 13:**
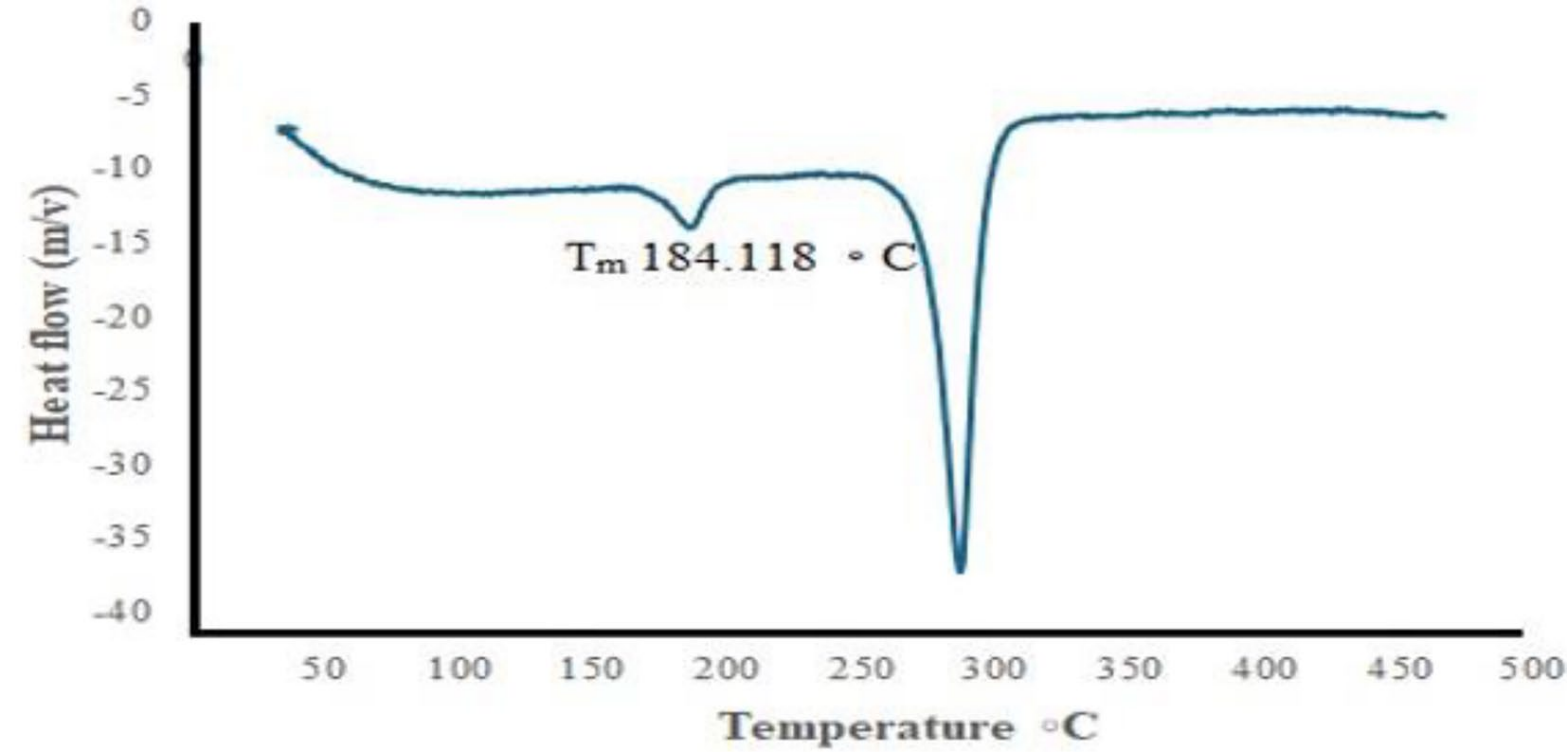
DSC investigated the PHB synthesized by *V. piezotolerans*.

Further analysis using DSC (Fig. 13) revealed that the extracted PHB exhibited a melting temperature (Tm) of 184.118 °C. This melting temperature is comparable to PHB obtained from other sources, such as EPPJ and glucose, which showed Tm values of 172 °C and 175 °C, respectively. These results are consistent with conventional PHB, which has a reported Tm of 176 °C^44^. The high thermal stability of polyhydroxyalkanoates (PHAs) is a critical factor for their polymerization processes, as it indicates the polymer’s ability to withstand elevated temperatures .

### Conclusion

In conclusion, the study successfully isolated and screened halophilic bacterial strains for polyhydroxybutyrate (PHB) production, identifying *Vreelandella piezotolerans* (MH 46) as the most effective producer using orange peel waste as the carbon source. Optimization experiments revealed that critical factors such as orange peel concentration, nitrogen source, and fermentation conditions significantly influenced PHB yield. Through both single-factor and response surface methodologies, optimal conditions were established, leading to enhanced PHB production. The characterization of the extracted PHB via GC-MS, FTIR, NMR, and XRD confirmed its structural integrity and thermal stability. These results underscore the potential of using agricultural waste for sustainable bioplastics, highlighting the role of microbial systems in addressing environmental challenges. Future research should focus on scaling up this process to promote broader industrial applications.

## Data Availability

Data availability statement: The datasets analyzed during the current study are available in the NCBI GenBank database repository with the accession number of PP826285 (https://www.ncbi.nlm.nih.gov/nuccore/PP826285).
